# Cirrhosis and Rapid Virological Response to Peginterferon Plus Ribavirin Determine Treatment Outcome in HCV-1 IL28B rs12979860 CC Patients

**DOI:** 10.1155/2013/580796

**Published:** 2013-07-09

**Authors:** Alessio Aghemo, Elisabetta Degasperi, Maria Grazia Rumi, Enrico Galmozzi, Luca Valenti, Raffaele De Francesco, Stella De Nicola, Cristina Cheroni, Eleonora Grassi, Massimo Colombo

**Affiliations:** ^1^Centro A.M. e A. Migliavacca, First Division of Gastroenterology, Fondazione IRCCS Ca' Granda Ospedale Maggiore Policlinico Milano, Università degli Studi di Milano, Via F. Sforza 35, 20122 Milano, Italy; ^2^Division of Hepatology, Ospedale San Giuseppe IRCCS MultiMedica, Università degli Studi di Milano, 20122 Milano, Italy; ^3^Division of Internal Medicine, Fondazione IRCCS Ca' Granda Ospedale Maggiore Policlinico, Università degli Studi di Milano, 20122 Milano, Italy; ^4^Istituto Nazionale Genetica Molecolare Milano (INGM), 20122 Milano, Italy

## Abstract

*Background*. The rs12979860 CC genotype of the interleukin 28B (IL28B) polymorphism is associated with high rates of sustained virological response (SVR) to peginterferon (PegIFN) and ribavirin (Rbv) in hepatitis C virus genotype-1 (HCV-1) patients. The impact of baseline predictors of treatment outcome and their interplay with viral kinetics in HCV-1 CC patients has not been fully evaluated. *Aim*. To identify baseline and on-therapy predictors of treatment failure in HCV-1 IL28B CC patients. *Methods*. Treatment-naïve HCV-1 patients, compliant to PegIFN and Rbv who did not discontinue treatment for nonvirological reasons, were analyzed. *Results*. 109 HCV-1 IL28B CC were studied. Sixty were males, 39 with BMI >25, 69 with >600,000 IU/mL HCV RNA, 15 with HCV1a, and 30 with cirrhosis. Overall, 75 (69%) achieved an SVR; cirrhosis was the only baseline predictor of treatment failure (OR: 2.58, 95% CI: 1.07–6.21) as SVR rates were 53% in cirrhotics versus 75% in noncirrhotics (*P* = 0.03). HCV RNA undetectability (<50 IU/mL) at week 4 (RVR) was achieved by 58 patients (53%). The SVR rates were independent of RVR in noncirrhotics, 76% (34/45) RVR (+) and 74% (25/34) RVR (−) (*P* = 0.9). In cirrhotic patients, SVR rates were significantly higher in RVR (+) compared to RVR (−) (10/13 (77%) versus 6/17 (35%) *P* = 0.03). *Conclusions*. In HCV-1 IL28B CC patients, cirrhosis is the only clinical baseline predictor of PegIFN and Rbv treatment failure. However, in IL28B CC cirrhotics, the achievement of RVR identifies those patients who still have high rates of SVR to Peg-IFN/Rbv therapy.

## 1. Introduction

Chronic infection with hepatitis C virus (HCV) affects almost 200 million people worldwide, representing a leading cause of cirrhosis and anticipated liver-related death [1]. Until 2011, treatment with pegylated interferon (PegIFN) and ribavirin (Rbv) was the standard of care (SOC) for all HCV genotypes, with HCV clearance depending both on virus and host-related factors [2]. In the last year, the significant improvement in sustained virological response (SVR) rates, attained by the addition of the first generation of directly acting antivirals (DAAs), the NS3 protease inhibitors Telaprevir or Boceprevir, to PegIFN plus Rbv in the difficult to cure HCV genotype 1 population, has created a new SOC for this group of patients [3–5]. However, there are doubts whether universal treatment of HCV-1 patients with a DAA-based regimen is cost-effective and safe, as DAAs including regimens are associated with new treatment-related side effects, some of which require expert management, many possible drug-drug interactions, and the risk of developing resistant viral strains [6–8]. In this scenario, the single nucleotide polymorphism (SNP) rs12979860 near the interleukin 28B (IL28B) region, which has been identified as the strongest baseline predictor of SVR to PegIFN plus Rbv in HCV-1 patients, has emerged as key factor in the pretherapy algorithm [9, 10]. Indeed, the high SVR rates obtained with PegIFN/Rbv in patients with the favorable IL28B genotype (CC) question the need for DAAs including regimens in these patients. Further supporting such an assumption is a recent Markov model cost-effectiveness analysis demonstrating that a DAA-based regimen does not provide added benefits over PegIFN/Rbv in this subgroup of patients [11]. For these reasons, some scientific societies have recommended that HCV-1 genotype IL28B CC patients be offered PegIFN and Rbv as a first-line therapy [12]. The only exemption being IL28B CC patients with cirrhosis, where a DAA-based regimen is considered the SOC by most guidelines due to a somewhat reduced efficacy of PegIFN and Rbv in these patients. However, such IL28B guided recommendations do not confer any clinical meaningfulness to other baseline moderators of treatment failure in IL28B CC patients nor do they value on-treatment viral kinetics in these patients. This is extremely significant as HCV RNA kinetics during treatment have been repeatedly shown to possess a stronger predictive power in terms of treatment outcome than any baseline pretreatment factor. With this as a background, we investigated HCV-1 IL28B CC patients treated with PegIFN plus Rbv to identify baseline and on-treatment predictors of treatment outcome.

## 2. Materials and Methods 

HCV-1 naïve patients who received PegIFN plus Rbv therapy in 5 clinical studies [13–17] conducted at the Liver Center and the Metabolic Center for Liver Disease of the Ospedale Maggiore Policlinico in Milan between 2003 and 2010 were scrutinized. All patients consented to genetic testing and were genotyped for the IL28B rs12979860 SNP. Those with CT or TT IL28B genotype and those with mixed HCV genotypes excluded from this analysis. Patients who discontinued PegIFN plus Rbv treatment for nonvirological reasons were not adherent to antiviral therapy, or received less than 80% of total PegIFN and Rbv expected doses were also excluded.

Chronic hepatitis C was defined by at least one year serum positivity for serum HCV-RNA, ALT levels >1.5 times the upper limit of normal, and by a liver biopsy, performed in the year preceding treatment, consistent with chronic hepatitis C. No patient was coinfected with hepatitis B virus (HBV) or human immunodeficiency virus (HIV) or had decompensated liver disease, drug dependence, or >20 g/day alcohol intake. Patients gave their written informed consent to receive therapy and to make their medical records available for this study which was approved by the Institutional Review Board of the Department of Internal Medicine. 

### 2.1. Measurements

Genomic DNA was obtained from whole blood or peripheral blood mononuclear cells using the QIAamp DNA Blood Mini Kit (Qiagen, Hilden, Germany). Genotyping of rs12979860 was performed using a 5′nuclease assay with allele-specific TaqMan probes, and assays were run on a 7900HT real time PCR instrument (Applied Biosystems, Carlsbad, CA, USA), following manufacturer's instruction or by using the tetra primer ARMS method [18, 19]. Serum HCV-RNA was assessed by qualitative RT-PCR assay (COBAS Amplicor HCV test version 2.0, Roche Diagnostics) with a detection limit of 50 IU/mL, during treatment at weeks 4, 12, 24, and 48 and after therapy at weeks 4, 12, and 24. Serum HCV-RNA was quantified at baseline and week 12 with Versant HCV-RNA 3.0 assay (bDNA 3.0, Bayer Corporation, Emeryville, CA), with a sensitivity limit of 615 IU/mL and a dynamic range from 615 to 7,700,000 IU/mL. HCV was genotyped by line probe assay (INNO-LIPA HCV 2, Innogenetics, Zwijndrecht, Belgium). Liver biopsies were performed with a 16 gauge Tru-Cut needle (Uro-Cut 16G, TSK, Tokyo, Japan) and read by a single pathologist, who was unaware of the patient's identity and treatment regimen. The severity of hepatic inflammation was evaluated by the Ishak score, which conferred a maximum of 18 points for grading (G) and 6 points for liver staging (S), so identifying incomplete and complete cirrhosis as S 5 and 6, respectively.

### 2.2. Treatment

Patients received Rbv (Rebetol, Schering-Plough Corporation, Kenilworth, NJ, USA) combined with either PegIFN*α*2a (Pegasys, Roche, Basel Switzerland) 180 mcg/week or PegIFN*α*2b 1.5 mcg/Kg/week (PegIntron, Schering-Plough Corporation) for 48 weeks. PegIFN*α*2a was associated with Rbv 1000–1200 mg day (<75 Kg; ≥75 Kg) while PegIFN*α*2b was associated with Rbv 800 mg for patients of less than 65 Kg body weight, 1000 mg for 65–85 Kg, and 1200 mg for ≥85 Kg. Therapy was discontinued if quantitative HCV-RNA testing at week 12 dropped by less than 2Logs compared to baseline values and at week 24 if HCV-RNA was still detectable in those patients in whom HCV-RNA dropped >2 Log at week 12. All patients were evaluated for safety and tolerance of treatment every 4 weeks during the treatment period. PegIFN*α*2a was reduced to 135 mcg and PegIFN*α*2b to 1.0 mcg/Kg per week in patients with <0.75 × 10^9^/L neutrophils at two consecutive tests, whereas it was withdrawn in patients with <0.50 × 10^9^/L. The same dose reductions were applied if platelets fell under 50,000 cells/mm^3^ with PegIFN being discontinued when reaching the 25,000 cells/mm^3^ threshold. In both treatment arms, Rbv dose was tapered by 200 mg/day in patients with haemoglobin <10 g/dL, whereas it was discontinued in patients with <8.5 g/dL haemoglobin. 

### 2.3. Definition of Response

Clearance of serum HCV-RNA by RT-PCR was assessed at week 4 (rapid virological response, RVR), at week 12 (complete early virological response, cEVR), at week 24, and at week 48 of treatment (end of treatment response, ETR). A sustained virological response (SVR) was undetectable HCV-RNA by RT-PCR at week 24 after treatment. Patients with an ETR who tested HCV-RNA positive during followup were classified as relapsers. Patients who had any other virologic response were considered as nonresponders.

### 2.4. Statistical Analysis

Comparisons between groups were made by using the Mann-Whitney *U* test or Student's *t*-test for continuous variables and the *χ* ^2^ or Fisher exact probability test for categorical data. A probability value of *P* < 0.05 was considered statistically significant. Logistic regression analysis was performed to identify the variables associated with PegIFN/Rbv treatment SVR. All variables with statistical significance at the univariate analysis were included in the final model, and odds ratios (OR) and corresponding 95% confidence interval (95% CI) were computed. Calculations were done with Stata 10.0 statistical package (Stata 1944–2007, College Station, TX, USA).

## 3. Results

Out of 409 HCV-1 patients enrolled in 5 clinical studies at two liver centers and meeting the inclusion criteria, 109 (27%) were IL28B CC and were included in the current analysis. The clinical and epidemiological characteristics of the 109 patients are shown in Table 1. 60 (55%) were males, the median age was 53 years (range 24–70), and 47 (43%) patients were older than 60 years. 39 (36%) patients had a BMI ≥25, and 30 (28%) had an histological diagnosis of cirrhosis (S 5, 6). Nine patients (8%) were obese due to a BMI >30. The prevalence of cirrhosis was independent of baseline BMI (BMI < 25: 17/70 (24%) versus BMI ≥ 25: 13/39 (33%), *P* = ns). HCV subtype was 1b in 94 (86%) and 1a in 15 patients (14%). 50 (46%) patients were treated with PegIFN*α*-2a, while 59 (54%) received PegIFN*α*-2b.

### 3.1. Baseline Predictors of Treatment Outcome

Overall 75 (69%) patients achieved an SVR. Of the 34 patients with a treatment failure, 3 (9%) were nonresponders while 31 (91%) had a posttreatment relapse. Although SVR rates did not significantly differ in the 5 clinical studies, they ranged from 64% to 76%. 

By univariate analysis, male gender, age, BMI > 25, HCV viral load, and HCV-1 subtype did not influence SVR rates, as the only baseline variable associated with treatment failure was the presence of cirrhosis (OR 2.58, 95% CI 1.07–6.21) (Table 2). Indeed, by stratifying SVR rates on the basis of liver staging, cirrhotic patients achieved significantly lower SVR rates than patients without cirrhosis ((16/30) 53% versus (59/79) 75%, *P* = 0.03). 

**Figure 1:**
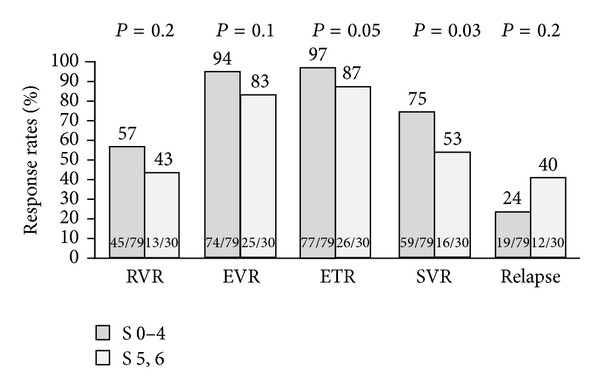
Treatment response rates stratified according to fibrosis stage.

Patients with cirrhosis showed numerically lower rates of RVR, EVR, and ETR and higher rates of posttreatment relapse compared to patients without cirrhosis (Figure 1). However, this difference was marginally statistically significant only for ETR rates (cirrhotics: 87% versus noncirrhotics: 97%, *P* = 0.05). 

### 3.2. On-Treatment Predictors

58 (53%) patients achieved an RVR, 99 (91%) an EVR, and 103 (95%) an ETR. Overall, the achievement of an RVR did not significantly affect the SVR rates, as the SVR rates were 76% in those with an RVR and 61% in those without an RVR (*P* = 0.1). 

**Figure 2:**
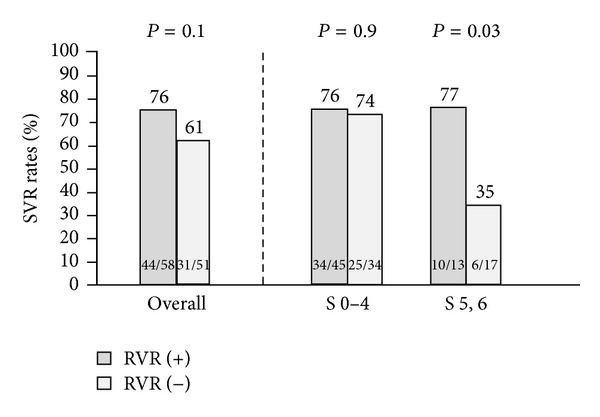
SVR rates stratified by fibrosis stage and RVR.

However, when stratifying patients by liver fibrosis staging, RVR emerged as a predictor of treatment outcome in those with cirrhosis. Indeed, in noncirrhotic patients the SVR rates remained homogenously high independently of the achievement of an RVR, 76% (34/45) in RVR (+) compared to 74% (25/34) in RVR (−) (*P* = 0.9). On the other hand, in patients with cirrhosis, the achievement of an RVR was associated with significantly higher SVR rates compared to those obtained in patients who failed to achieve an RVR, SVR being, respectively, 77% (10/13) in RVR (+) and 35% (6/17) in RVR (−) (*P* = 0.03), hence identifying RVR as a strong predictor of SVR in these patients (OR 6.1, 95% CI: 1.2–31.2) (Figure 2).

## 4. Discussion

Our study shows that cirrhosis is the only baseline predictor of treatment failure in HCV-1 IL28B CC patients, with 53% of cirrhotic patients achieving an SVR compared to 75% of noncirrhotics. Our data do not only partially replicate a previous subanalysis of the IDEAL study, showing lower SVR rates in IL28B CC patients with bridging fibrosis and cirrhosis [10], but add nicely to that study by showing the importance of on-treatment viral kinetics in these patients. The achievement of an RVR in cirrhotic patients was associated with high rates of SVR (77%), which contrast with the low SVR rates (35%) in cirrhotic patients who were still HCV RNA positive after 4 weeks of treatment. This is a novel finding since several studies have shown RVR to lose its strong predictive power in IL28B CC patients of any HCV genotype [20, 21]. Indeed, although CC patients are more prone than patients with the T allele to achieve an RVR, the SVR rates remain high also in the subgroup of CC patients that do not reach this endpoint. This was recently magnified by a study analyzing the benefit of treatment extension to 72 weeks in HCV-1 and 4 slow responders, which reported no significant increase in SVR rates in IL28B CC patients mainly as a direct cause of the satisfactory SVR rates obtained by the standard 48-week treatment regimen [22]. Our study confirms this observation but only in those without cirrhosis, as in this subgroup the SVR rates were independent of RVR status. Although this finding might be at least in part related to the low sensitivity of our HCV RNA assay, this data nicely replicates findings from a previous study using an HCV RNA assay with an LOD of 27 IU/mL [10].

On the other hand, RVR did play a key role in predicting treatment outcome in IL28B CC patients with cirrhosis, as SVR rates were surprisingly low in those not reaching this virological landmark. Although we cannot provide a definitive explanation for this interplay, it is probable that, in the setting of an altered liver architecture with a deranged microcirculation due to fibrosis deposition [23], which might impair PegIFN/Rbv antiviral effect [24], the achievement of an RVR identifies a subgroup of patients still able to activate the complex pathways necessary to define an antiviral state, which ultimately results in persistent viral eradication also in the presence of cirrhosis.

While this hypothesis further reinforces the dominant role of RVR over pretreatment variables as the most powerful predictor of SVR, our characterization of the interplay between fibrosis staging and on-treatment kinetics has important clinical implications. Since a universal migration to DAA-based regimens in all HCV-1 patients might not be affordable in terms of healthcare resources in countries where a welfare state is in place, correct selection of patients for a DAA-based regimen is mandatory. In Europe, most, local guidelines suggest offering HCV-1 patients with the IL28B CC genotype, PegIFN/Rbv as the first-choice therapy, while offering a DAA-based regimen only to IL28B CC patients with advanced fibrosis/cirrhosis [6, 12, 25, 26]. From this point of view, our findings suggest the need for better stratification in cirrhotic patients, as this subset of patients likely represents the most prone to suffer from side effects with DAA regimens, does not benefit from response guided treatment duration with these drugs, as cirrhotic patients require in all cases 48 weeks of treatment, and ultimately might be potentially more harmed by the development of drug resistances. Based on our results, the combination of IL28B genotype and HCV-RNA testing after 4 weeks of treatment could improve the decision making process, as a four-week PegIFN/Rbv lead-in phase in IL28B CC cirrhotic patients could discriminate those requiring the addition of a DAA from those who could continue with dual therapy. Our proposal in theory would contrast with the fact that only boceprevir has been registered with a lead-in phase, de facto limiting this new algorithm to just one of the new DAAs; still several expert opinions suggest that a lead-in phase can be safely and effectively applied to telaprevir regimens too [3–5, 27]. While in theory such an approach would seem cost effective over universal treatment with DAAs, it needs to be validated in a cost effectiveness analysis taking into account indirect costs such as IL28B testing and availability as well as fibrosis staging through liver biopsy or noninvasive methods.

We acknowledge that our study has several limitations; first of all it is retrospective, and it includes only patients who were adherent to the optimal treatment schedule and were enrolled in 5 clinical studies where both PegIFNs were administered. Given the different efficacy of the two regimens reported by us and others [13, 28] and the different sensitivity to degree of fibrosis reported in some studies [24], this calls for external validation of our results in large cohorts of patients receiving both PegIFNs. Moreover, due to the extended enrolment period, the PCR assay used in our study for on-treatment serum HCV-RNA assessment had a relatively high limit of detection (50 IU/mL) that could have misclassified some RVR patients and suggests validation of our results with an HCV RNA assay with a lower detection limit [29, 30]. Lastly, in our study cirrhosis was histologically defined, a practice that is common in clinical trials but less so in everyday clinical practice, due to the reliability of noninvasive methods to assess liver fibrosis (i.e., transient elastography) [31]. However, since noninvasive methods are known to be negatively influenced in terms of accuracy and reproducibility by being overweight or obese, something that we reported in 36% of our cohort of patients, liver biopsy is likely to remain essential in any treatment-related algorithm guided by fibrosis staging [32]. Most importantly, our patients with cirrhosis were all Child-Pugh A5, had in all cases a platelet count >75 × 10^3^/mm^3^ (range 90–260 × 10^3^/mm^3^), and had esophageal varices in just 1 case (3%), stressing the concept that our data apply only to well-compensated cirrhotics without clinical stigmas of portal hypertension.

Notwithstanding these limitations, our demonstration of an interplay between cirrhosis and RVR in IL28B CC patients provides evidence for individualized treatment algorithms also in this subgroup of highly responsive patients.

## Figures and Tables

**Table 1:** Baseline characteristics of the 109 HCV-1 rs12979860 CC patients.

Patients	Overall (*N* = 109)
Males	60 (55%)
Age, years (mean)	53 (range 24–70)
Weight, kg (mean)	68.4 (41–110)
BMI > 25	39 (36%)
Liver fibrosis stage (Ishak)	
S 0–2	50 (46%)
S 3-4	29 (26%)
S 5-6	30 (28%)
HCV genotype	
1b	94 (86%)
1a	15 (14%)
Baseline serum HCV-RNA	
>0.6 × 10^6^ IU/mL	69 (63%)
PegIFN type	
alfa2a	50 (46%)
alfa2b	59 (54%)

**Table 2:** Epidemiological and clinical characteristics stratified by treatment outcome.

	SVR (*n* = 75)	Non-SVR (*n* = 34)	*P* value*	OR for non-SVR (95% CI)
Male sex, *n* (%)	41 (55%)	19 (56%)	1	1.05 (0.46–2.37)
Age, years (median)	56	60	0.19	1.02 (0.99–1.07)
BMI > 25, *n* (%)	24 (32%)	15 (44%)	0.29	1.66 (0.73–3.86)
HCV-1a, *n* (%)	13 (18%)	2 (6%)	0.13	0.29 (0.06–1.40)
High viral load, *n* (%)°	44 (59%)	25 (85%)	0.2	1.95 (0.80–4.76)
Cirrhosis, *n* (%)	16 (21%)	14 (41%)	0.03	2.58 (1.07–6.21)
PegIFN alfa-2a, *n* (%)	38 (51%)	12 (41%)	0.15	0.53 (0.23–1.22)

*Mann-Whitney *U* test for continuous variables, Fisher's exact test for categorical variables.

°Baseline serum HCV RNA > 0.6 × 10^6^ IU/mL.

## Abbreviations

HCV: Hepatitis C virus
IL28B: Interleukin 28B
SVR: Sustained virological response
PegIFN: Peginterferon
Rbv: Ribavirin
RVR: Rapid virological response
DAA: Directly acting antivirals
SNP: Single nucleotide polymorphism
HBV: Hepatitis B virus
HIV: Human immunodeficiency virus
cEVR: Complete early virological response
ETR: End of treatment response
OR: Odds ratios
95% CI: 95% confidence interval.
